# The Actin Cytoskeleton Is Involved in Glial Cell Line-Derived Neurotrophic Factor (GDNF)-Induced Ret Translocation into Lipid Rafts in Dopaminergic Neuronal Cells

**DOI:** 10.3390/ijms18091922

**Published:** 2017-09-07

**Authors:** Li Li, Haijing Song, Peipei Mu, Ming Xu, Chaoxia Liu, Ying Wang, Yingsong Qin, Shen Sun, Jin Gao, Ting Wang, Dianshuai Gao

**Affiliations:** 1 Department of Pathophysiology, School of the Basic Medicine, Xuzhou Medical University, Xuzhou 221004, Jiangsu, China; xzmclili@163.com (L.L.); xzmcxm@163.com (M.X.); xzmclcx@163.com (C.L.); xzmcwy@163.com (Y.W.); xzmcqys@163.com (Y.Q.); 2 Institute of Emergency Rescue Medicine, Xuzhou Medical University, Xuzhou 221004, Jiangsu, China; xzmcshj@163.com; 3 School of Nursing, Xuzhou Medical University, Xuzhou 221004, Jiangsu, China; xzyxympp@163.com; 4 Department of Histology and Embryology, School of the Basic Medicine, Xuzhou Medical University, Xuzhou 221004, Jiangsu, China; 5189009@xzhmu.edu.cn; 5 Department of Neurobiology and Anatomy, Xuzhou Key Laboratory of Neurobiology, Jiangsu Key Laboratory of New Drug Research and Clinical Pharmacy, Xuzhou Medical University, Xuzhou 221004, Jiangsu, China; xzmcgj@163.com; 6 Institute of Medical Technology, Xuzhou Medical University, Xuzhou 221004, Jiangsu, China; wt27518@sina.com

**Keywords:** GDNF, Ret, dopaminergic cells, lipid rafts, cytoskeleton

## Abstract

Glial cell line-derived neurotrophic factor (GDNF), a potential therapeutic factor for Parkinson’s disease (PD), exerts its biological effects through the Ret receptor tyrosine kinase. The redistribution of Ret into lipid rafts substantially influences Ret signaling, but the mechanisms underlying Ret translocation remain unclear. The purpose of our study was to further explore the signaling mechanisms of GDNF and to determine whether the actin cytoskeleton is involved in the GDNF-induced Ret translocation into lipid rafts. In MN9D dopaminergic neuronal cells, we used density gradient centrifugation and immunofluorescence confocal microscopy to separate and visualize lipid rafts, co-immunoprecipitation to analyze protein-protein interactions, and latrunculin B (Lat B) and jasplakinolide (Jas) to disrupt and enhance the polymerization of the actin cytoskeleton, respectively. The results showed that Ret translocated into lipid rafts and coimmunoprecipitated with actin in response to GDNF treatment. After Lat B or Jas treatment, the Ret–F-actin association induced by GDNF was impaired or enhanced respectively and then the levels of Ret translocated into lipid rafts were correspondingly inhibited or promoted. These data indicate that actin polymerization and cytoskeletal remodeling are integral to GDNF-induced cell signaling in dopaminergic cells and define a new role of the actin cytoskeleton in promoting Ret redistribution into lipid rafts.

## 1. Introduction

Parkinson’s disease (PD) is a common neurodegenerative disease characterized by degeneration of dopaminergic neurons in the substantia nigra. Glial cell line-derived neurotrophic factor (GDNF), a member of the transforming growth factor (TGF)-β superfamily, has been shown to promote the survival of dopaminergic neurons in midbrain and serve as a promising neuroprotective therapy for PD [1,2,3].

GDNF acts through the receptor tyrosine kinase Ret, which plays important roles in cell differentiation, survival, and migration during embryonic development. Previous studies have demonstrated that in response to GDNF, Ret translocates to cholesterol-rich membrane microdomains known as lipid rafts, and the proper membrane localization of Ret is fundamentally important for GDNF signaling [4,5,6,7]. However, the specific mechanisms underlying the translocation of Ret between membrane compartments (from non-lipid rafts to lipid rafts) under GDNF treatment remain incompletely understood.

Lipid rafts are enriched in cholesterol and sphingolipids and can serve as essential signaling platforms by concentrating signal transduction molecules in the cell membrane [8,9,10]. In recent years, factors that may direct the formation of lipid rafts continue to be elucidated. One such factor is the actin cytoskeleton. The actin cytoskeleton is composed of a network of filaments that underlie and attach to the plasma membrane. Recent data have shown that the actin cytoskeleton can affect the lateral distribution and clustering of rafts proteins in the membrane and may play an important role in the formation of membrane rafts [11,12]. Furthermore, the cytoskeleton also regulates the lateral diffusion of non-raft component proteins in response to extracellular events [13,14].

However, whether the actin cytoskeleton participates in GDNF-induced translocation of Ret between different membrane compartments has not been established.

In this study, we utilized murine mesencephalic cell line, MN9D, as a model of dopaminergic (DA) neuronal cells. MN9D is a hybridoma cell line derived from the fusion of primary embryonic cells from the mouse ventral midbrain with the neuroblastoma cell line N18TG2. There are a number of important similarities between these cells and DA neurons and MN9D is extensively used to model DA neuronal cells [15,16]. We used density gradient centrifugation and immunofluorescence confocal microscopy to separate and visualize lipid rafts, co-immunoprecipitation to analyze protein–protein interactions, and latrunculin B (Lat B) and jasplakinolide (Jas) to disrupt and enhance the polymerization of the actin cytoskeleton, respectively. The results showed that in response to GDNF stimulation, Ret translocated to lipid rafts and co-immunoprecipitated with F-actin. After Lat B or Jas treatment, the Ret–F-actin association induced by GDNF was impaired or enhanced respectively and then the levels of Ret translocated into lipid rafts were correspondingly inhibited or promoted. These data illustrate that actin polymerization and cytoskeletal remodeling are integral to GDNF-induced cell signaling in dopaminergic cells and define a new role of the actin cytoskeleton in promoting Ret redistribution into lipid rafts.

## 2. Results

### 2.1. Differentiation of MN9D Neuronal Cells

**Figure 1:**
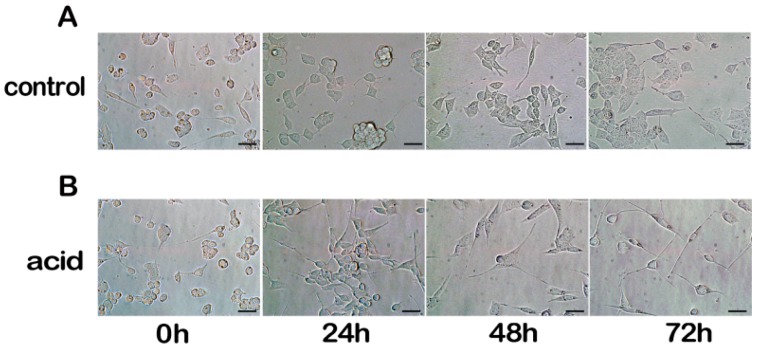
Differentiated and undifferentiated MN9D cells. MN9D cells were observed by fluorescence microscopy at 0, 24, 48, and 72 h after plating. (**A**) Undifferentiated MN9D cells were plated in complete growth medium; (**B**) MN9D cells were plated in complete growth medium plus 1 μM retinoic acid. (Scale bar = 20 μm).

In this study, MD9D cells were differentiated by using previous method [16]. The MN9D cells were differentiated in complete medium with 1 μM retinoic acid for 3 days (Figure 1).

### 2.2. GDNF Induces Ret Translocation to Lipid Rafts

**Figure 2:**
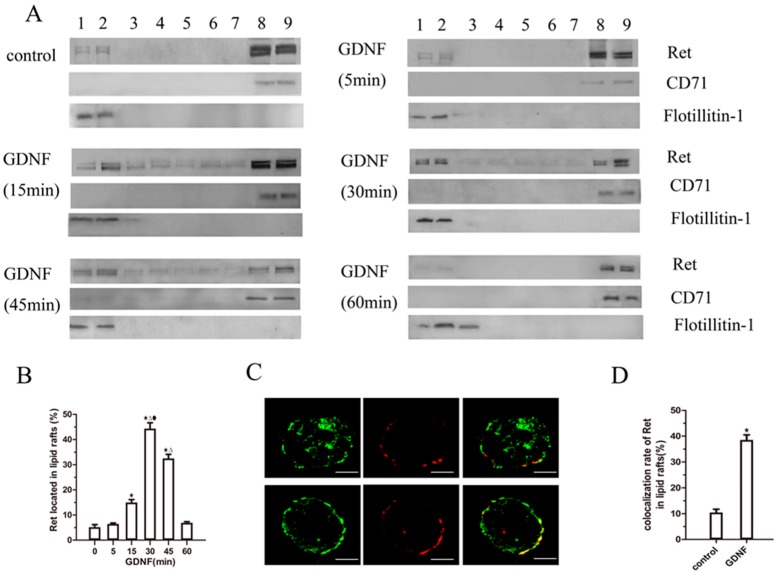
Ret associates with lipid raft fractions after GDNF stimulation. (**A**,**B**) Differentiated MN9D cells were treated with medium alone or with GDNF (50 ng/mL) for 5, 15, 30, 45, and 60 min, respectively. The cells were then lysed and subjected to OptiPrep™ density centrifugation as described in the methods. Nine equal fractions were obtained and labeled from 1 to 9. These fractions were analyzed by western blotting for Ret, flotillin-1, and CD71. The data were represented as means ± SEM of three independent experiments. ^★^ *p* < 0.05 vs. 0 min, ^Δ^ *p* < 0.05 vs. 15 min, ^●^ *p* < 0.05 vs. 45 min; (**C**,**D**) Differentiated MN9D cells were treated with medium alone or with GDNF (50 ng/mL) for 30 min. Then lipid rafts (red) and Ret (green) patching was induced as described in the methods. Confocal microscopy was used to detect the colocalization of lipid rafts and Ret. The data were represented as means ± SEM of three independent experiments. ^★^ *p* < 0.05 vs. 0 min. (Scale bar = 5 μm).

To confirm that GDNF induced Ret translocation to lipid rafts, we examined the subcellular localization of Ret protein in MN9D cells by OptiPrep™ density gradient centrifugation. In the absence of GDNF stimulation, only minimal Ret immunoreactivity was identified in the raft microdomains (fractions 1–2); the raft-associated protein Flotillin-1 was also detected in this fraction. The most Ret immunoreactivity was detected in a non-raft fraction (fractions 8–9), and a characteristic non-raft-associated protein, CD71, was also detected in this fraction. After GDNF stimulation, the Ret levels in the lipid rafts gradually increased and reached a maximum at approximately 30 min. Ret translocation began to decline at approximately 45 min. At 60 min, the localization of Ret had returned to baseline levels (Figure 2A,B).

To further confirm GDNF-induced Ret translocation into lipid rafts, we used patching and immunofluorescence to visualize the colocalization of lipid rafts and Ret after GDNF treatment for 30 min. We found that ganglioside GM1 was patched after CT-B/anti-CT-B treatment, and only minimal Ret patches were colocalized with CT-B patches in the absence of GDNF. Stimulation with GDNF for 30 min resulted in increased colocalization of Ret and CT-B patches (Figure 2C,D). These results indicated that Ret was preferentially localized to glycosphingolipid-rich domains after GDNF stimulation.

### 2.3. GDNF Induces the Association of Ret and F-Actin

**Figure 3:**
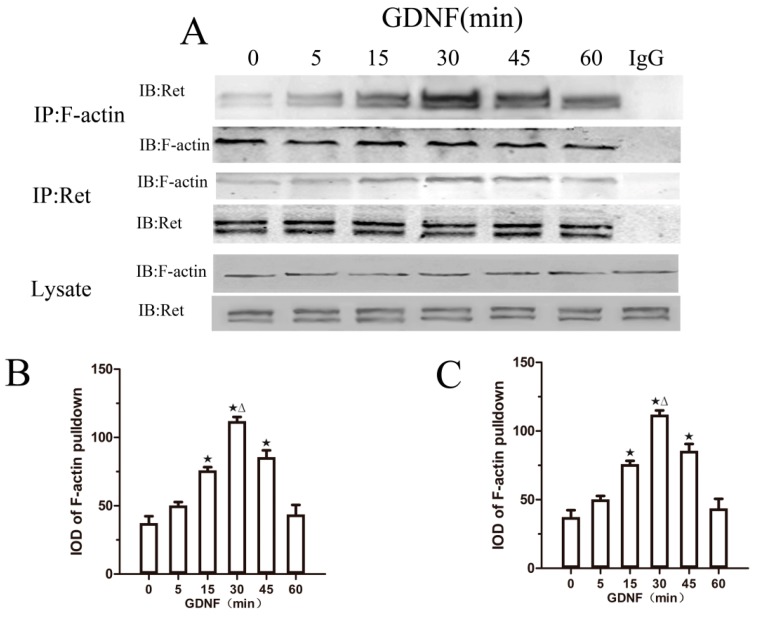
GDNF induces Ret–F-actin association in cultured MN9D cells. (**A**) Lysates obtained from MN9D cells, which were stimulated with GDNF for the indicated durations, were immunoprecipitated (IP) with anti-F-actin, anti-Ret, or normal rabbit IgG (IgG IP). As a loading control, the amount of F-actin and Ret present in the whole lysates is shown at the bottom; (**B**) Quantitative analysis of integrated optical density (IOD) of immunoprecipitated Ret from the experiments are depicted in (**B**); (**C**) Quantitative analysis of IOD of immunoprecipitated F-actin from the experiments are depicted in (**C**). Data were presented as the mean ± SEM of three independent experiments. ^★^ *p* < 0.05 vs. 0 min, ^Δ^ *p* < 0.05 vs. 15 min.

To confirm whether F-actin is involved in GDNF-mediated Ret translocation to lipid rafts, we performed co-immunoprecipitation experiments. In the absence of GDNF stimulation, we detected very little association between Ret and F-actin. After 5 min of GDNF treatment, there was a small increase in the Ret–F-actin association that became more pronounced at 15 min and peaked at approximately 30 min. After 30 min, the levels of co-immunoprecipitated Ret–F-actin declined but were still higher than the levels without GDNF treatment. Additionally, when we used anti-Ret to co-immunoprecipitate F-actin in the presence of GDNF, similar results were observed (Figure 3). Our findings suggest that GDNF induces an association between Ret and F-actin.

### 2.4. Lat B and Jas Disrupt and Enhance the Polymerization of the Actin Cytoskeleton, Respectively

**Figure 4:**
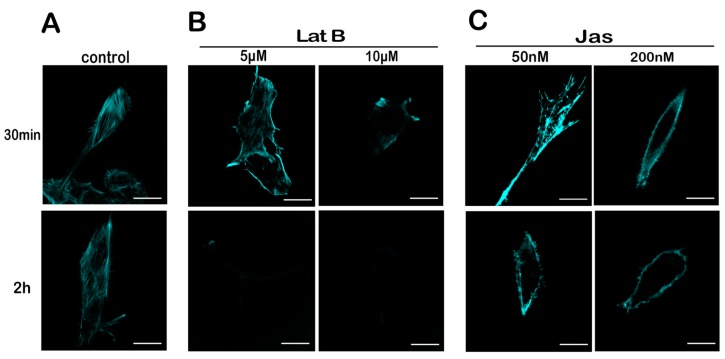
The concentration-dependent effects of Lat B and Jas on the actin cytoskeleton. (**A**) Untreated MN9D cells were stained with phalloidin at the given time points; (**B**) MN9D cells were stimulated with Lat B at the indicated concentrations and time points; (**C**) MN9D cells were stimulated with Jas at the indicated concentrations and time points. (Scale bar = 5 μm).

To screen concentration and time of Lat B or Jas treatment, MN9D cells were treated with Lat B (5 μM, 10 μM) or Jas (50 nM, 200 nM) for 30 min or 2 h, respectively. After the cells were treated with 5 μM Lat B for 30 min, there is no obvious loss of the structure of the actin in the cells. When the Lat B concentration was increased to 10 μM, very little actin staining was seen, thus indicating impaired actin polymerization. After a 2 h exposure to 5 μM or 10 μM Lat B, actin became difficult to detect (Figure 4B). When the cells were treated with 50 nM Jas for 30 min, there is an increase in the fluorescence intensity of F-actin. However, when the cells were exposed to 50 nM Jas for 2 h and 200 nM Jas for 30 min or 2 h, F-actin was almost completely depleted in the central region of the cells with actin staining observed only at the cell margins (Figure 4C). On the basis of these results, we selected 10 μM Lat B and 50 nM Jas for 30 min as our working concentrations and time.

### 2.5. Lat B and Jas Regulate the Association between Ret and F-Actin

**Figure 5:**
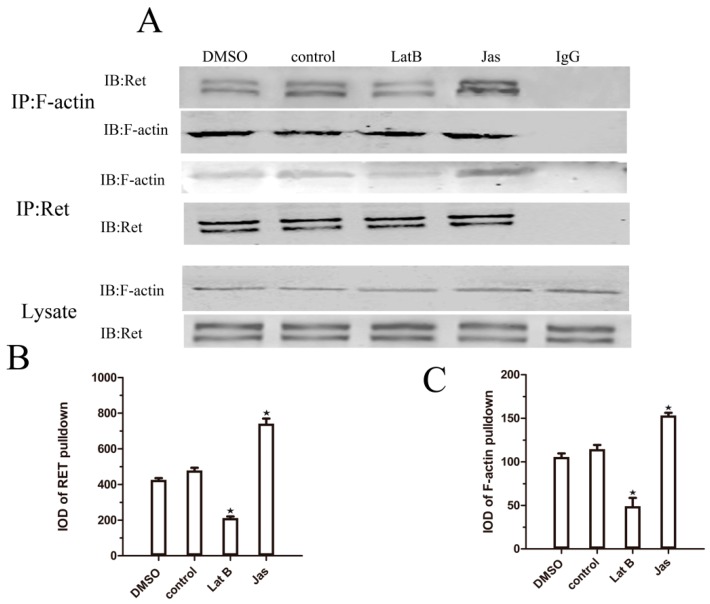
GDNF-induced Ret–F-actin association is impaired by Lat B or promoted by Jas. (**A**) The MN9D cells were divided into the following four groups: in the control group, the cells were cultured under normal conditions and treated with GDNF for 30 min; in the dimethylsulfoxide (DMSO)group (solvent group), the cells were pretreated with 1 mM DMSO for 30 min and then treated with GDNF for 30 min; in the Lat B group, the cells were pretreated with 10 μM Lat B for 30 min and then treated with GDNF for 30 min; and in the Jas group, the cells were pretreated with 50 nM Jas for 30 min and then treated with GDNF for 30 min. Lysates obtained from MN9D cells were immunoprecipitated with anti-Ret, anti-F-actin, and normal rabbit IgG. As a loading control, the amount of Ret and F-actin present in the whole lysates is shown at the bottom; (**B**) The quantitative analysis of IOD of co-immunoprecipitated Ret from the experiments is depicted in (**B**); (**C**) The quantitative analysis of IOD of co-immunoprecipitated F-actin from the experiments is depicted in (**C**). The data were represented as means ± SEM of three independent experiments. ^★^ *p* < 0.05 vs. control.

To confirm whether the GDNF-induced association between Ret and F-actin is affected by Lat B and Jas, we performed co-immunoprecipitation experiments after GDNF treatment for 30 min. First, lysates were prepared and subjected to immunoprecipitation using anti-F-actin. With GDNF stimulation, the level of associated Ret–F-actin was increased in accordance with the co-immunoprecipitation results mentioned above. However, the level of Ret co-immunoprecipitated with F-actin was decreased when the cells were treated with Lat B and was increased when the cells were treated with Jas (Figure 5). No changes were observed when the lysates were immunoprecipitated with normal rabbit IgG. When using the Ret antibody to immunoprecipitate, we obtained the similar results. This result suggested that the GDNF-induced association of Ret with lipid rafts is dependent on the regulation of actin polymerization.

### 2.6. The Actin Cytoskeleton Is Involved in GDNF-Induced Ret Translocation into Lipid Rafts

**Figure 6:**
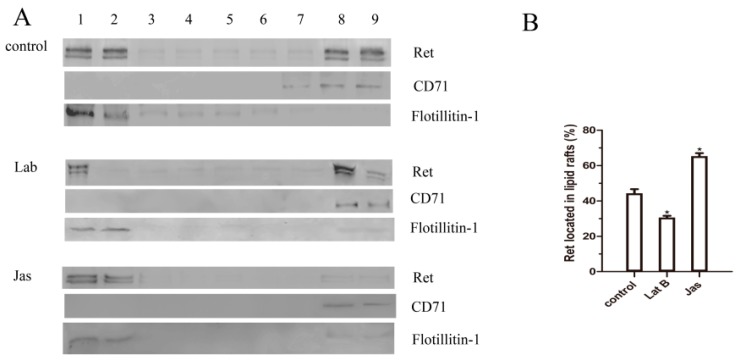

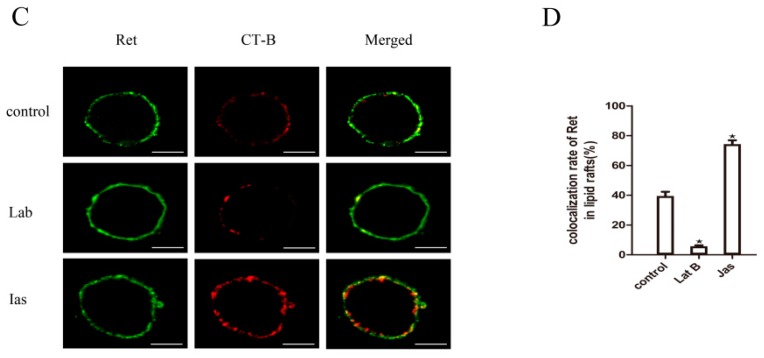
The effects of Lat B and Jas on Ret translocation to lipid rafts. The cells were divided into three groups. The control group was treated with GDNF only for 30 min; the Lat B group and the Jas group were pretreated with Lat B or Jas for 30 min respectively, and then treated with GDNF for 30 min. (**A**,**B**) The cells were then lysed and subjected to OptiPrep™ density centrifugation. Nine equal fractions were obtained and labeled from 1 to 9. These fractions were analyzed by western blotting for Ret, flotillin-1, and CD71; (**C**,**D**) Then lipid rafts(red) and Ret(green) patching was induced as described in the methods. Confocal microscopy was used to detect the colocalization of lipid rafts and Ret. The data were represented as means ± SEM of three independent experiments. ^★^ *p* < 0.05 vs. control. (Scale bar = 5 μm).

To confirm that the GDNF-induced translocation of the Ret into lipid rafts is dependent on the actin cytoskeleton, the cells were divided into three groups, and all groups were treated with GDNF for 30 min. The control group was treated with GDNF only, and the Lat B or Jas group were pretreated with Lat B or Jas, respectively, for 30 min before GDNF treatment. After Lat B pretreatment, GDNF-induced Ret translocation was reduced, and Ret mainly remained in the non-raft fractions with CD71. In contrast, the Jas group exhibited substantial translocation of Ret to lipid rafts. This result demonstrated that Lat B disrupted the binding of actin to Ret, which in turn decreased the translocation of Ret into lipid rafts. In contrast, the Jas group exhibited substantial translocation of Ret to lipid rafts (Figure 6A,B). These results indicated that actin polymerization was integral to GDNF-induced Ret translocation.

We observed similar results with patching and immunofluorescence confocal microscopy. Lat B treatment reduced the colocalization of Ret with lipid rafts. In contrast, the colocalization of Ret with lipid rafts was abundant in the Jas group (Figure 6C,D). These data provide additional evidence that the GDNF-induced association of Ret with lipid rafts is dependent on the actin cytoskeleton.

## 3. Discussion

GDNF is a member of the TGF-β superfamily and was first identified as a potent pro-survival factor for midbrain dopaminergic neurons. GDNF as a promising neuroprotective factor for PD transduces neuroprotective signal via Ret receptor tyrosine kinase, which translocates into lipid rafts after GDNF treatment.

Lipid rafts are membrane microdomains that are rich in cholesterol and sphingolipids and form more ordered lipid bilayers than the surrounding plasma membrane [8,9,10]. In addition, many signaling molecules, such as glycerophospholipid (GPI)-anchored proteins, G protein-coupled receptors, receptor tyrosine kinases, and signaling proteins with saturated acyl chains are localized to lipid rafts [17,18]. Raft microdomains may compartmentalize groups of signaling molecules and allow them to interact with each other within rafts, while preventing them from interacting with molecules outside the rafts. Therefore, lipid rafts may function as specialized signaling platforms in the plasma membrane and are therefore critical for signal transduction regulation [19,20,21].

In our study, in response to stimulation with GDNF, the levels of Ret in lipid rafts increased and reached a maximum at 30 min. This is in accordance with previous studies [4,5,6,7]. However, the mechanisms for directing Ret distribution between these membrane microdomains remained incompletely understood.

In recent years, factors that may direct the formation of lipid rafts continue to be elucidated. One such factor is the actin cytoskeleton. The actin cytoskeleton is composed of a network of filaments that underlie and attach to the plasma membrane. The actin cytoskeleton is particularly enriched under the inner surfaces of lipid rafts and co-associates with known membrane raft markers [22,23,24,25]. In addition, a variety of cytoskeletal components (actin, tubulin, vinculin, filamin, and tau) have been detected in lipid rafts, thus suggesting a close link between lipid rafts and the cytoskeleton [26,27].

Recent data have shown that the actin cytoskeleton can affect the lateral distribution and mobility of raft component proteins in the membrane. For example, Chichili and Rodgers have described that actin is involved in co-clustering of membrane raft-associated proteins [11]. Moreover, actin remodeling and polymerization have been demonstrated to regulate the lateral movement of GPI-anchored protein and ultimately lead to clustering of GPI-anchored proteins in raft fractions [12]. In addition, the cytoskeleton also can regulate the lateral diffusion of non-raft component proteins in response to extracellular events. For instance, evidence has indicated that actin-driven clustering of rafts drive compartmentalization of T-cell receptor (TCR) in to lipid rafts [13,14]. Sun et al. has also found that oscillatory shear stress (OS) induced integrin α5 translocation into lipid rafts depend on the F-actin-based cytoskeleton in human umbilical vein ECs (HUVECs) [28].

However, whether the actin cytoskeleton is involved in GDNF-induced Ret translocation into lipid rafts is unclear. Using co-immunoprecipitation we found that GDNF could induce interactions of Ret and actin cytoskeleton. After Lat B and Jas treatment, the interactions were disrupted and promoted respectively and then the levels of Ret translocated into lipid rafts were correspondingly inhibited or promoted.

Our present study indicates that in response to GDNF stimulation, Ret translocated to lipid rafts in the MN9D cells. Furthermore, the remodeling of the actin cytoskeleton is integral to GDNF-induced cell signaling in dopaminergic cells, defining a new role of the actin cytoskeleton in promoting Ret redistribution into lipid rafts. Determining the mechanisms of Ret translocation not only promotes an understanding of the protective mechanisms of GDNF on dopaminergic neurons, but also provides novel insights into the movement of membrane signaling proteins and the dynamic assembly of lipid rafts.

However, from the present data, we cannot conclude whether Ret is directly tethered to actin filaments or indirectly linked through intermediate proteins. Therefore, in future studies, we will focus on the specific and detailed mechanisms underlying the interaction between Ret and the actin cytoskeleton.

## 4. Materials and Methods

### 4.1. Cell Culture and Differentiation Assay

MN9D cells were grown on 25 cm^2^ culture flasks (Corning, Corning, NY, USA) at a density of 5 × 10^5^ in complete medium comprising DMEM/F12 (HyClone Laboratories, Inc., Logan, UT, USA), 10% fetal bovine serum (Gibco, Grand Island, NY, USA), and 1% penicillin/streptomycin (Beyotime Biotechnology Co., Beijing, China). The cells were maintained in a water-jacketed incubator at 37 °C under 5% CO_2_. One day after plating, the medium was changed to complete medium plus 1 μM retinoic acid (Sigma-Aldrich, St. Louis, MO, USA), and the cells were incubated for at least 3 days before the experiments.

### 4.2. Isolation of Detergent-Resistant Membrane Fractions and Analysis of Membrane Fractions on Flotation Gradients

All steps were performed on ice in a cold room at 4 °C MN9D cells were grown in 25 cm^2^ culture flasks and stimulated with 50 ng/mL GDNF (Sigma-Aldrich) for the indicated times at 37 °C. Then, the cells were washed twice with phosphate buffered solution (PBS) and scraped into the medium. The cell pellet was resuspended in 670 µL isolation buffer (150 mM NaCl, 5 mM DTT, 5 mM EDTA, 25 mM Tris-HCl, and a protease inhibitor cocktail). After the cells were homogenized by 20 passages through a 22G syringe needle, the homogenate was centrifuged at 1000× *g* for 10 min. Then, 1% Triton X-100 was added to the supernatant, which was incubated on ice for 30 min. After the incubation, we added 40% OptiPrep™ to the homogenate, which we accomplished by first adding 1330 µL isolation buffer to the bottom of a 12 mL SW41Ti ultracentrifuge tube (Beckman Coulter, Palo Alto, CA, USA). We then added additional layers of homogenate mixed with 2 mL of 35, 30, 25, and 20% OptiPrep™ in isolation buffer and added 2 mL of isolation buffer to fill the tube. After centrifugation (18 h, 200,000× *g*, 4 °C), nine equal fractions were taken from the top of the tubes and labeled from 1 to 9, with fraction 9 being the densest. These fractions were analyzed by western blotting for Ret (1:1000, Abcam, Cambridge, MA, USA), flotillin-1 (marker of lipid raft, 1:100, Santa Cruz Biotechnology, Dallas, TX, USA), and CD71 (marker of non-raft fractions, 1:100, Santa Cruz Biotechnology). To estimate the percentage of raft associated Ret versus total Ret, we normalized the immunoblot intensity of the Ret bands in the raft fractions (lanes 1–3) to the total Ret (lanes 1–9).

### 4.3. Patching and Immunofluorescence Confocal Microscopy

Lipid raft patching was induced as described by Fra et al. and Peter et al. with slight modifications [29,30]. Briefly, for all fluorescence microscopy experiments, the cells were attached to 35 mm poly-l-lysine-coated dishes at a density of 2 × 10^5^. Cholera toxin subunit B (CT-B) labeled with Alexa Fluor 594 (Molecular Probes Inc., Eugene, OR, USA) was used at 10 mg/mL in PBS to label ganglioside GM1, which selectively partitions into lipid rafts. An anti-CT-B antibody (1:200 in PBS; Molecular Probes Inc.) was used to crosslink the CT-B-labeled lipid rafts into distinct patches on the plasma membrane. The cells were then incubated for 10 min and 15 min at 4 °C. The presence of lipid raft aggregation or patching was confirmed after CT-B and anti-CT-B labeling. For fixation, the cells were directly treated with 4% paraformaldehyde in PBS for 15 min.

Ret patching was induced in the same manner. The cells were incubated with a primary anti-Ret antibody (1:1000, Abcam) overnight at 4 °C, and this was followed by incubation with an Alexa Fluor 488-conjugated goat anti-rabbit IgG secondary antibody (Molecular Probes Inc.) for 2 h at room temperature. Confocal microscopy (FV10-ASW, Olympus, Tokyo, Japan) was performed with a 60× oil immersion objective, using laser excitation at 350, 488, and 594 nm.

### 4.4. Ret/F-Actin Co-Immunoprecipitation

Ret was immunoprecipitated from control, DMSO-, 10 μM latrunculin B- (Lat B; QCC, CA, USA), 50 nM jasplakinolide- (Jas; QCC, CA, USA) or GDNF-treated MN9D cell lysates. After treatment, the cells were washed in cold PBS and harvested in lysis buffer (50 mM HEPES, 150 mM NaCl, 1.5 mM MgCl_2_, 1 mM ZnCl_2_, 1 mM EGTA, 10% glycerol, 1% Triton X-100, 50 mM NaF, 20 mM sodium pyrophosphate, 1 mM PMSF, and 5 mg/mL each of NA3VO4, leupeptin, aprotinin, and pepstatin). Cleared lysates were assayed for protein content and normalized to total protein. Then, the lysates were immunoprecipitated with 3 µg/100 µg anti-Ret antibody and a mixture of Protein A/Protein G agarose conjugates (Pierce Biotechnology, Thermo Fisher, Rockford, IL, USA), and then subjected to SDS-PAGE analysis. The protein was transferred onto a nitrocellulose filter (NC) membrane, and blots were probed with appropriate antibodies and detected with HRP-conjugated secondary antibodies. The antibodies used for western blotting were as follows: Ret and F-actin (1:100, Santa Cruz Biotechnology).

### 4.5. Western Blotting

The cells were lysised in RIPA buffer (50 mM Tris, 150 mM NaCl, 1% Triton X-100, 1% sodium deoxycholate, 0.1% SDS, sodium orthovanadate, sodium fluoride, EDTA, and leupeptin) and centrifuged to yield whole-cell lysates. A Bradford protein concentration assay was used to measure the total protein concentration. Aliquots of the lysates (20 μg of protein) were separated on a 5–10% SDS-polyacrylamide gel with SDS running buffer (25 mM Tris (pH 8.3), 250 mM glycocoll, and 0.1% SDS) and transferred onto a nitrocellulose filter (NC) membrane (Invitrogen, Carlsbad, CA, USA) with transfer buffer (25 mM Tris-HCl (pH 8.3), 192 mM glycocoll, and 20% methyl alcohol). Then the membrane was incubated with specific primary antibodies and IR Dye^®^800 anti-rabbit or anti-mouse secondary antibodies (Li-Cor, Lincoln, NE, USA) sequentially. Immunoreactive proteins were detected using an Odyssey^®^ Imaging System (Li-Cor). Band intensities were quantified using Image-Pro Plus software (Media Cybernetics, Rockville, MD, USA).

### 4.6. Statistical Analysis

All data are reported as the mean ± SEM. Statistical significance between multiple groups was assessed using one-way analysis of variance (ANOVA), and differences between groups were determined with Scheffe’s *t*-test. A *p*-value less than 0.05 was considered significant.

## 5. Conclusions

Ret translocated to lipid rafts in the MN9D cells in response to GDNF stimulation. The remodeling of the actin cytoskeleton is integral to GDNF-induced cell signaling in dopaminergic cells, defining a new role of the actin cytoskeleton in promoting Ret redistribution into lipid rafts.
